# Antiretroviral therapy and changing patterns of HIV stigmatisation in Entebbe, Uganda

**DOI:** 10.1111/1467-9566.12341

**Published:** 2015-09-18

**Authors:** Steve Russell, Flavia Zalwango, Stella Namukwaya, Joseph Katongole, Richard Muhumuza, Ruth Nalugya, Janet Seeley

**Affiliations:** ^1^ School of International DevelopmentUniversity of East AngliaNorwichNorfolk; ^2^ Medical Research CouncilUganda Virus Research InstituteUganda Research Unit on AIDSUganda

**Keywords:** HIV, stigma, chronic illness, developing countries, doctor–patient communication/interaction, gender

## Abstract

Antiretroviral therapy (ART) has the potential to change processes of HIV stigmatisation. In this article, changing processes of stigmatisation among a group of people living with HIV (PLWH) on ART in Wakiso District, Uganda, are analysed using qualitative data from a study of PLWH's self‐management of HIV on ART. There were 38 respondents (20 women, 18 men) who had been taking ART for at least 1 year. They were purposefully selected from government and non‐government ART providers. Two in‐depth interviews were held with each participant. Processes of reduced self‐stigmatisation were clearly evident, caused by the recovery of their physical appearance and support from health workers. However most participants continued to conceal their status because they anticipated stigma; for example, they feared gossip, rejection and their status being used against them. Anticipated stigma was gendered: women expressed greater fear of enacted forms of stigma such as rejection by their partner; in contrast men's fears focused on gossip, loss of dignity and self‐stigmatisation. The evidence indicates that ART has not reduced underlying structural drivers of stigmatisation, notably gender identities and inequalities, and that interventions are still required to mitigate and tackle stigmatisation, such as counselling, peer‐led education and support groups that can help PLWH reconstruct alternative and more positive identities.

A video abstract of this article can be found at: https://youtu.be/WtIaZJQ3Y_8

## Introduction

### Background and rationale

HIV combines all the characteristics of stigmatised medical conditions (Alonzo and Reynolds 1995). It is incurable and fatal, contagious, a threat to the life of others, physically degenerative and disfiguring, and associated with a painful or unaesthetic death. More profoundly, HIV is often seen to pose a threat to the moral values and social order of the community (Ogden and Nyblade 2005). It is associated with morally disapproved of behaviour, especially that expected of women, and contracting HIV is viewed as the responsibility of the individual, exposing people to blame and judgement. The progression of stigma follows the progression of the condition; as people become visibly more sick, stigma and discrimination increase (Alonzo and Reynolds 1995).

The increased uptake of antiretroviral therapy (ART) in resource‐limited countries over the last 10 years has meant some of these stigmatising characteristics are potentially being reduced as disease progression is halted or reversed, profoundly changing the social experience of that disease (Castro and Farmer 2005). With ART HIV has become more like a manageable chronic condition rather than a death sentence; people can look well and can work again and may get back to their ‘normal’ lives.

Early studies in resource‐limited settings indicated that ART could reduce internalised and enacted stigma, for example, in Haiti (Castro and Farmer 2005), and in South Africa, where people's recovery on ART had encouraged them to be open about their status and become activists in campaigns for access to treatment (Robins 2006). However, subsequent studies reveal more complex and mixed findings, with stigma persisting in sub‐Saharan Africa and continuing to act as a barrier to testing or disclosure (Blackstock 2005, Mbonu *et al*. 2009, Simbayi *et al*. 2007, Wolfe *et al*. 2006). More specifically, the following patterns appear to be emerging. Firstly, ART enables a process of reduced self‐stigmatisation among PLWH (Campbell *et al*. 2011, Gilbert and Walker 2009, Mbonye *et al*. 2013, Roura *et al*. 2009a, 2009b). Secondly, ART is not changing, or will be slower to change, the underlying causes of stigma; notably moral discourses that judge and blame women and men for transgressing the rules or norms of the social order, which means people still fear, anticipate and experience stigma from others (Bond 2009, Genberg *et al*. 2009, Maughan‐Brown 2010, Mbonye *et al*. 2013, Roura *et al*. 2009a, 2009b, Simbayi *et al*. 2007). This is perhaps not surprising, given the well‐developed theoretical literature on stigmatisation which sees it being driven not just by the visible attributes of a person but by social processes which label groups and link them to prevailing undesirable characteristics in that social and moral setting (Link and Phelan 2001).

In this article we present qualitative findings about the changing nature of HIV stigma among a group of PLWH on ART in Uganda following the introduction of ART, to add to the evidence base and to consider the implications for people's self‐management of HIV, as well as for interventions to address stigma.

Uganda is an interesting context in which to examine processes of stigmatisation following the introduction of ART, because it was one of the first African countries to expand access to the treatment in the early 2000s (Seeley 2014). ART is now widely available, especially in the study setting of Wakiso District around Kampala and Entebbe, and many PLWH have been taking ART for several years. Initiatives to tackle stigma were also introduced at an early stage of the epidemic, notably through the work of non‐governmental organisations such as the AIDS Support Organisation (TASO), which provided counselling services and helped PLWH establish support groups to mitigate self‐stigmatisation (Ssebbanja 2007).

## Theoretical frameworks

Theoretical frameworks that explain stigmatisation as a social process are now well established (Castro and Farmer 2005, Deacon 2006, Link and Phelan 2001, Mahajan *et al*. 2008, Parker and Aggleton 2003, Phelan *et al*. 2008, Wyrod 2013), and usually begin with Goffman's (1963: 3) work, which defined stigma as ‘an attribute that is deeply discrediting’. The theoretical frameworks then critique subsequent work that focused on stigma as an individual characteristic of a person. Link and Phelan (2001) note that Goffman (1963) related stigmatising attributes to socially produced stereotypes, and in their theory of stigma as a social process, differences between groups are socially constructed and affixed to others by processes of labelling. Labelled differences are linked to negative attributes and stereotypes, making the difference undesirable or tainted in a given social and moral setting: for example, being HIV positive is equated with being promiscuous, irresponsible, untrustworthy and a danger to others. By putting people into categories of difference, a separation or distinction between ‘them and us’ can be made; stigmatised groups can be inserted into structures of power and inequality, and can be discriminated against.

These stigmatisation processes derive from and are dependent on social inequalities such as those based on gender, class or ethnicity, because they involve the exertion of power by dominant groups over subordinate groups. Stigma then operates to reproduce these inequalities, by labelling negatively and punishing groups that deviate from the social norms which sustain inequality (norm enforcement). Stigma serves the purpose of increasing conformity with social norms, clarifying to people ‘the boundaries of acceptable behaviour … and the consequences for non‐conformity’ (Phelan *et al*. 2008: 362). Stigma therefore polices the moral and social order, acting as ‘a mechanism for sharpening the boundaries of the ‘moral community,’ between ‘us’ (the normal/righteous/upstanding citizens) and ‘them’ (the deviant/bad/’fallen’ ones)’ (Ogden and Nyblade 2005: 22).

Norm enforcement is linked to a second function of stigma that reproduces inequality; namely power and domination (keeping people down) (Phelan *et al*. 2008). By discrediting a person's and group's moral integrity, stigma legitimises their low position in the social hierarchy, and enables them to be placed in systems of discrimination, social exclusion and disadvantage (Link and Phelan 2001, Parker and Aggleton 2003, Phelan *et al*., 2008).

Patterns of stigmatisation examined in this article are analysed with a focus on gender relations and how these shape men's and women's experiences of stigma. The links between gender and stigmatisation can be analysed at structural, interpersonal and intra‐personal levels (Wyrod 2011, 2013). At the structural level, in any society there are socially constructed norms and rules about men and women's ‘nature,’ and how they should think, behave and interact. These deeply embedded structures are sometimes formally institutionalised into laws and organisational forms and procedures. At an interpersonal level, these structures shape people's roles and relationships and their daily interactions. Through these actions men and women reproduce these structures, but their agency also means they can negotiate, challenge and change them. At the intra‐personal level, gender norms, values and notions of how a man or woman should behave become internalised and naturalised.

Stigma can also be understood using this three dimensional framework. At the intra‐personal level there is internalised or self‐stigma, when a person internalises the prevailing values in the wider community which judge and label them negatively, diminishing their sense of self and self‐esteem which can cause self‐exclusion. At the interpersonal level people experience acts of verbal or physical discrimination or enacted stigma by others. Our analysis also refers to anticipated stigma, which derives from both intra‐personal (self) stigma and fears of interpersonal (enacted) stigma, defined as ‘the reaction people expect from others if it were to become known that they were living with HIV’ (Roura *et al*. 2009b: 4). The structural level is the main basis or driver of self, enacted and anticipated stigma.

Gendered hierarchies mean that across societies, women usually experience greater levels of HIV stigmatisation than men (Castro and Farmer 2005, Ogden and Nyblade 2005). In South Africa and Swaziland, for example, Campbell *et al*. (2006) and Shamos *et al*. (2009) show how HIV stigma is deployed more against women, who are expected to be responsible for and uphold sexual morality, and so are more easily blamed and harshly judged for any sexual transgressions that HIV is socially constructed to embody or represent. Stigmatisation therefore reproduces patriarchal power structures that seek to suppress and control women's sexuality (Campbell *et al*. 2006). HIV stigmatisation is a mechanism of policing, used to discipline and punish those who challenge norms or informal rules that legitimise existing structures of control over women (Campbell *et al*. 2006).

More specific constructions of femininity in any given setting also shape HIV stigma, for example in Tanzania, moral discourses that stigmatise HIV are particularly targeted at women migrants returning from the city due to constructions of femininity that expect ‘proper’ women not to be economically independent or alone in the city, and their sexuality to be under the surveillance and control of men (Dilger 2008). Their migration to the city for work, where their freedoms and sexuality cannot be controlled, challenges these patriarchal hierarchies. In Uganda there is a similar, long‐standing moral discourse that negatively labels and stigmatises town women who live and work in urban areas and earn their own money (Davis 2000, Ogden 1996). This construct derives from colonial times when women migrated to the city (Kampala) and sold domestic services or provided sex to male migrant workers. A negative construction of town women as sex workers arose from ‘othering’ processes which could use simple dichotomies of town or single women versus rural or married women; the latter category being proper women who follow gender rules by being submissive to and economically dependent on their husbands (Davis 2000). This negative construction and stereotyping of some poor urban women continues, despite economic and social changes that mean it is quite normal for women to be working in urban areas in many different forms of employment or business (Davis 2000, Ogden 1996). In the era of HIV, women with HIV can be easily blamed for transgressing the rules of sexual behaviour for proper women and be linked to this broad, stigmatised social category of town women (Davis 2000).

Constructions of gender generate substantial privileges for men, but men also experience HIV stigma through the hierarchies of masculine identity (Wyrod 2011, 2013). The notion of hegemonic masculinity is used by Wyrod (2011) to explain why men living with HIV may experience stigma; notably self‐stigmatisation. General characteristics of hegemonic masculinity include physical strength, being resilient in the face of challenges, self‐reliance and sexual prowess and dominance over women. In the Ugandan context, Siu *et al*. (2013) and Wyrod (2011) note the following specific signifiers of hegemonic masculinity: having sexual partners, producing children and being a successful breadwinner who can provide for his family (responsible fatherhood). Men with HIV experience intra‐personal (self) stigmatisation in particular, because an HIV diagnosis can undermine their ability to do or be these key dimensions of hegemonic masculinity: such men may feel they no longer embody or enact normative masculine identities, and cannot be like a proper man due to an incapacitating condition like HIV (Wyrod 2011).

Coping with stigma is an important dimension of HIV self‐management (Swendeman *et al*. 2009), involving adjustment to a new sense of self and decisions about disclosure. Self‐management of one's HIV status and identity in the community, for example, through what Goffman (1963) termed concealment strategies, is an understandable response to anticipated stigma and the real threat of enacted stigma.

## Methods

### Research design and study site

The qualitative data presented in this article were collected in 2011–2012 as part of a study in Wakiso District on the coping and self‐management processes of PLWH on ART. Three types of ART delivery site in the district were selected to recruit participants: the HIV clinic at the government hospital in Entebbe, three government health centres that have referral links to Entebbe hospital and the Entebbe branch of a well‐established non‐governmental organisation, TASO.

Ethical approval for the study was obtained from the Uganda Virus Research Institute Science and Ethics Committee and the International Development Research Ethics Committee, University of East Anglia, UK. Overall permission to conduct the research was obtained from the Uganda National Council for Science and Technology. Written informed consent was obtained from all participants in the research. Pseudonyms are used in this article to maintain confidentiality.

To be eligible participants must have been on ART for more than 1 year. Eligible patients were listed for each facility, and a systematic random sample taken using intervals to generate twice the number of cases required. These lists were then stratified by age and gender, and 42 participants were purposively sampled from gender and age categories to ensure a gender balance and a mix of ages. Four could not be interviewed successfully or more than once and were excluded from final analysis.

### Data collection measures

The participants in this study were interviewed twice. The first interview was an unstructured life and illness history interview, conducted over one to three visits due to the wide‐ranging nature of the questions. To help people feel more comfortable and open in their responses, these interviews were not recorded but notes were taken and detailed narratives were written up in English by the interviewers.

The second interview was semi‐structured, and this was recorded, transcribed and translated into English. The question guide was informed by issues raised in the life history interviews as well as the research objectives and theoretical frameworks. The guide included questions about stigmatisation and disclosure. The use of several visits to meet participants allowed a degree of trust and rapport to develop, which in many cases led to rich discussions of participants’ experiences.

### Analysis

Qualitative data were organised and initially analysed using QSR Software NVivo 9. To check the rigour of analysis, two researchers independently did the initial coding and checked results. Themes and sub‐themes were identified based on the narrative content, the research questions and the theoretical frameworks on illness self‐management and stigmatisation informing the research. Thematic interpretations of the data were discussed and agreed by the team at a 2‐week analytical workshop held in Entebbe, Uganda in 2012. Themes were tested by checking counter examples and exceptions. Quotes used in the article are either the words of the participants or the interviewer's words used in the write‐up of the first interview. Frequently repeated expressions used by participants are not quoted but cited using italics.

## Results

### Participants’ characteristics

Table 1 summarises the socio‐demographic characteristics of the 38 participants, of whom 13 were from Entebbe hospital, 11 from the three referral health centres and 14 from TASO Entebbe.

**Table 1:** Sample for the qualitative study (*N* = 38)

Age (years)	Male	Female	Total (n, %)
0–17	0	0	0 (0)
18–25	0	2	2 (5)
26–40	10	10	20 (53)
41–60	7	7	14 (37)
61+	1	1	2 (5)
Total	18	20	38 (100)

More than half of the participants had some primary education and most were married or in a relationship. They mainly engaged in subsistence farming, fishing, building and petty trade. Nearly half the participants were income poor (8/18 men; 10/20 women), and with differing frequency struggled to meet basic food needs. Those who were able to cultivate around their homes were usually able to eat one meal a day, but a small minority in extreme income poverty faced a daily struggle for enough food. For many, therefore, HIV stigmatisation would be experienced in conjunction with the stigma of being poor.

### Reduced self‐stigmatisation

At the time they fell sick or were diagnosed, to different degrees all the participants experienced self‐stigma, internalising moral discourses that associated HIV with promiscuity, irresponsibility and a discredited feminine or masculine identity. Men, in particular, expressed the shame they had felt for being unable to work and having to rely on others when they fell sick. Several participants, especially the women, had experienced enacted stigma, usually in the form of verbal insults or being physically isolated, which reinforced self‐stigmatisation:

I cried and I felt that I had turned into a disgusting thing, because the way my aunt talked [when seeing me at home] was as if she had found faeces.

(Sarah, Female (F), aged 38)

Some people would refuse to eat and drink when I was around.

(Naome, F, 26)

After counselling and beginning ART, processes of reduced self‐stigmatisation were evident in all the narratives, although some of the men and women had to work much harder than others to cope with self‐stigma, and a few still felt a deep sense of shame about their status. Most participants spoke about their renewed self‐esteem, positive outlook and acceptance of their condition. Several interrelated processes contributed to reduced self‐stigmatisation.

Firstly, healthcare providers played an important role in helping PLWH come to terms with their condition, understand it and reconceptualise or reframe the illness to reconstruct a positive identity (Watkins‐Hayes *et al*. 2012). Counselling provided participants with concepts and language that ‘normalised’ HIV, seeing it as a treatable and manageable disease, like many other diseases*,* rather than a death sentence:

Things have changed [HIV is more accepted] … now it is as if it is like any other disease, like you see sickle cell, pressure, ulcers or cancer.

(Tom, M, 44)

Reconceptualising HIV as a normal disease helped participants reappraise their identity as a normal person. HIV was also framed as a normal disease through reference to its prevalence in the community. Health workers had told them from the start *you are not alone, just look around you*. All the participants drew on this language: ‘We are very many on drugs in this area … we are many and they admire us’ (Grace, F, 32).

The participants soon saw that they shared a problem with many others because of the crowds at TASO or the government clinic. Reduced self‐stigmatisation was also shaped by positive interactions with and peer support from fellow patients at the clinic. These interactions created a sense of belonging, membership and solidarity at the clinic, which for many was a space where new friends, support networks and new positive identities were forged.

Secondly, the reconceptualisation of HIV encouraged resistance thinking against stigmatisation, and health workers encouraged this resistance thinking by providing them with a language of comparison between themselves and *the many others who had not gone for a test and were ignorant of their status*. They could view themselves, individually and as a group, as knowledgeable and responsible; they had taken action to get tested, gain control, and were not harming others:

They [the health workers] told us that we were better than those who had not bothered to know their status, that we were better than those that were laughing at us. They laugh at you, saying that the [TASO] motorcycle has come to your home, yet they are also sick but do not take the responsibility to go and get tested so they don't know their status. That is what made me brave.

(Judith, F, 27)

Thirdly, the participants’ self‐esteem was strongly reinforced as their physical health and bodily appearance improved on ART. A powerful theme in the narratives was their joy about *looking* better, for passing as normal in the community, which enhanced self‐esteem and facilitated a return to social activity:

It's unfortunate that I don't have any photos near here, but in those days when I had just tested, I would fear to sit in a congregation or I would feel small whenever I would meet with other people. But nowadays I no longer care because I don't carry any sign of HIV. I don't care being looked at. But in the past I used to be suspicious whenever someone looked at me because I had lost weight and used to cough … I would think ‘they are saying I am infected’.

(Ruth, F, 58)

Some participants who were more open about their status were proud of how well they looked:

In the past there was so much fear [about HIV] … . [But now] I drink my beer and I tell the people around that I am HIV infected, and I am proud … I show off because I look good.

(Mark, M, 31)

Fourthly, participants’ ability to do productive work again or look after their children, and the fact that they were not incurring high treatment costs, reduced stigma related to dependency or burden. Participants were working hard, and for many this was also part of getting back to normal, interacting with colleagues and others in the community. Their life was no longer defined by their illness. Men took pride in regaining their physical strength and being able to provide for their families again, embodying and signifying a return to normal or proper masculinity. Single women also took pride in their ability to provide for their families again.

### Anticipated stigma

A small number of participants, especially women, still experienced enacted stigma after starting treatment. For example, Ruth described her sister's reactions: ‘from the time she got to know my status she has found me nauseating and will not share items’ (Ruth, F, 58). A far more prominent theme, however, was that ART *had* changed attitudes because people no longer looked sick, causing a reduction in enacted stigma:

They say that HIV is now like fever because ARVs are available … *sirimu* [the word for HIV in Luganda, meaning thin] means losing weight but people no longer lose weight so they are not afraid of it.

(Paloma, F, 31)

A common phrase describing this process was *people are now sharing our cups and plates*. Despite nearly all participants saying ART had changed attitudes, only a few were open about their status in the wider community, saying an open approach was good for their wellbeing: they had put their fear to one side, no longer had to worry about people finding out, and were keen to play a role in raising awareness:

Park your MRC vehicle outside my shop, I am not afraid of what others think. I do not hide my status from anyone – it is up to them if they want to still see me.

(Grace, F, 32)

Most participants, however, still anticipated the possibility of stigma, which undoubtedly reflected some degree of continuing self‐stigmatisation as well as fears of enacted stigma. Participants therefore adopted a range of concealment behaviours as part of their self‐management. These included choosing to go to the hospital clinic rather than joining TASO, because being seen at TASO was a clear public statement of your HIV status, and travelling further from home to pick up their drugs (two participants). Others strongly requested that the fieldworkers’ vehicle should not come to their home, and one female participant demanded that the vehicle be parked at least 2 kilometres away; some concealed their pill taking in public. One of the health centres recognised the threat of stigma to the uptake of HIV services in their locality:

It's only the health workers who know about our status and when people who are positive go to the health centre they mix us up with other patients so no one can tell and this has helped many to access treatment without fear … if they had separated them, they would not have gone to the health centre.

(Nana, F, 46)

Non‐disclosure was the main identity management strategy discussed by participants, especially men. At one extreme, one man had told only one family member ‘his secret’, and only once, and had not even told his medical companion the condition for which he took drugs.

Others had been slightly more open, and told their partner or a few members of their family, but even those relatively more open about their status limited disclosure to their close family (parents, siblings), a few relatives such as an uncle or aunt, a few close friends and occasionally, an employer. Disclosure to new intimate partners remained problematic, especially for women (see below).

Selective disclosure was a response to previous experiences and observations of stigmatisation. They feared, or at least could not predict, how people would react:

You cannot know who your true friend is. You may tell someone and instead of helping you, she just talks about you and she asks how you got infected.

(Ritah, F, 39)

A closely related theme of secrecy and silences about HIV was also evident:

Let us lower our voices; I do not want my daughter to know I have HIV.

(Bridget, F, 33)

When she was diagnosed she told her sister, who comforted her, and her sister then said that she herself was HIV positive and went to TASO. Her sister had not talked about it with her all this time.

(Judith, F, 27)

Judith had not disclosed to her neighbours, but the TASO motorbike gave her away. She was very hurt by her status becoming known, and this forced her to be open with her neighbours. At the point when she told her neighbours, they did not disclose to her, but kept their secret until Judith bumped into them at TASO.

(Judith, F, 27)

He has not disclosed to his wife … but suspects that she knows because of the drugs she sees him take.

(Jerry, M, 45)

Several participants could not bring themselves to tell their loved ones, even though they were confident they would not be rejected, because they could not bear to change forever how they would be perceived. Dorcas, for example, a schoolteacher, was too scared to tell her eldest daughter, her brother and her boss (headmaster), even though she accepted they probably knew she was HIV positive. Her boss probably knew, for example, because his wife is positive and they saw each other at the clinic. Paloma summed up this feeling well: ‘My heart won't let me (reveal)’ (Paloma, F, 31).

Two broad categories of fear about others knowing could be distinguished: (i) fears relating to self‐stigmatisation; notably fear of gossip and visible side‐effects of ART that could threaten one's own sense of worth and dignity in the community, and (ii) fear of enacted stigma; notably fear of rejection and fear of one's status being used against you. These different types of anticipated stigma were, to some degree, patterned by gender. Women expressed fears about self‐stigma and enacted stigma if their status was revealed; in contrast men more commonly focused on their fears about loss of dignity and self‐stigmatisation, rather than enacted stigma, fearing what others in the community would think and say about them, and wanting to keep it secret to preserve their dignity.

### Fear of gossip and visible side‐effects of ART

Fear of what people would say about you when you were not present was the most common and strongly emphasised reason for careful management of disclosure in the community. Both women and men expressed their fear of gossip, but for men this was their main fear: they feared losing their masculine status and dignity among other men and women in the community, especially male peers and work colleagues:

In this village, aaa! I cannot tell them, I remain here like a king, and I do not spend time sympathising with myself, or that everywhere I pass worry about people thinking I am sick and I am going to die.

(Davis, M, 43)

But I do not go around talking about my status. I still preserve my dignity. So when my time for taking drugs comes, I do not take them amongst people … We do not know most people's thoughts or behaviour.

(Benson, M, 34)

They say so much about us, for example, they say that you are sick and when you walk or are even seen talking to someone of the opposite sex they say that you are going to infect them with HIV.

(Bridget, F, 33)

Participants heard gossip at work, at relatives’ homes and at funeral gatherings, and said people gossiped to identify others in the community with HIV:

People still gossip: they talk about HIV because it is a hot topic, they even point at those with HIV … They investigate, then gossip about us.

(Bridget, F, 33)

Even if you look healthy, they still have to talk and make sure everybody knows you are infected. (Sarah, F, 38).

People talk about ‘so and so being infected; don't you see the way they are now and how they have changed’. People use their eyes to check you.

(Derrick, M, 38)

The degree to which participants anticipated and feared gossip varied, and a few had developed resistance strategies:

People's talk in the community is insulting. When they find out you are taking drugs, they say ‘that *ka* one is sick’ [a derogatory term meaning the person has been reduced to nothing or something small] … But you keep on walking because those who are talking do not know their HIV status and they may be sick without knowing.

(Ann, F, 29)

Passing as normal had considerably enhanced wellbeing. Yet the relief in knowing that nobody could tell said as much about ongoing fears of people finding out and potential stigmatisation. Participants remained highly sensitised to their bodily appearance:

At times I get to think that probably people look at me and they are able to tell my HIV status but again I get it off my mind as soon as possible.

(Sarah, F, 38)

Participants believed that if the disease became more visible, stigma would again be enacted against them, implying they felt the underlying causes of stigma remained but were latent. As the length of time on ART increases, the visible side‐effects of ART become more likely, such as darkened skin and nails or a change in body shape. These side‐effects are an alternative mark that people feared could give away their status. Women in particular were sensitised to visible side‐effects:

[Due to the drugs] some of her body parts had become thin and had protruding veins … her arms had been well balanced with her body size but now she is looking funny because they appear smaller … She feels so bad because she is losing shape, and … when somebody who is knowledgeable about ART sees her, s/he can just tell that she is infected and on drugs.

(Dorcas, F, 42)

### Fear of rejection and HIV ‘being used against you’

Female participants, in particular, feared a strong negative reaction or outright rejection by family members, friends, colleagues, employers and, in particular, new intimate partners. Women were more fearful about disclosing to a new partner than men. One female participant abandoned treatment for 1 year because she had migrated with a new partner whom she had not told. She could not travel back to obtain her drugs because she feared he would find out. In another case, a participant who was pregnant from a new partner felt too scared and vulnerable to disclose: ‘If I had told my husband I was sick he would have thrown me out, but I was pregnant and needed support’ (Joy, F, 27).

Women's greater fear of rejection by a male partner stemmed from their greater material vulnerability if the relationship should end and the greater risks to them of violence, both resulting from stronger enacted sanctions against women who are seen to have transgressed rules of proper female honour and sexual conduct. However, some men with new partners did also struggle to disclose, but mainly for their own dignity, rather than fears of enacted stigma.

Non‐disclosure to friends, work colleagues and employers was more commonly mentioned by men, and explained as a decision to help retain their job, their dignity at work and a good social life among fellow men. Signifiers of masculine identity in this context, notably toughness and the ability to work, explain why men were so reluctant to disclose among work colleagues:

I don't want that [others to talk about me]. I want them to remain among my friends.

(Isaac, M, 38)

I think that the head teacher knows that I am infected with HIV because I always ask for permission to go to the health centre on ART clinic days but I have never told her my HIV status. Other than this, I have not told any of the other teachers about my status.

(Aaron, M, 40)

Participants also did not disclose because they feared their status would be used against them during a conflict or argument. More women expressed a fear of this type of enacted stigma than men:

Although they [my family] are proud of me, they still at times abuse me saying I have AIDS.

(Naome, F, 26)

When somebody gets to know you have a problem, he or she provokes you and in case you react, he or she abuses you saying that you have AIDS.

(Nana, F, 46)

The participants feared that others would assume moral superiority in an argument and use this as a weapon to undermine their moral integrity and put down any arguments or claims they made.

## Discussion

The findings show continuing and changing processes of stigmatisation in the era of ART. Evidence of reduced self‐stigmatisation among men and women was compelling, although some men and women still struggled to deal with this issue. Patterns of difference across genders were also apparent, notably for different forms of anticipated stigma.

Reduced self‐stigmatisation has been found in other studies from the region (Gilbert and Walker 2009, Mbonye *et al*. 2013, Roura *et al*. 2009a, 2009b), caused by several interrelated processes: the recovery of health and a normal physical appearance, returning to productive activities and effective support and care from health workers. Health workers substantially helped participants to reconceptualise or reframe HIV as a normal disease and to resist stigmatising discourses, processes documented elsewhere (Watkins‐Hayes *et al*. 2012).

The participants were helped to reconstruct their identities as knowledgeable and responsible citizens, compared to ‘the others’ who were ignorant of their status. This ‘them and us’ construct helped generate a sense of belonging and collective identity among participants, building their confidence and self‐esteem. A sense of solidarity was further built by making friends and having positive interactions with fellow patients at the clinic. The positive effects of in‐group belonging for self‐esteem have been well demonstrated in the psychology literature (Yalom and Leszcz 2005).

Participants’ return to physical health on ART and passing as normal was fundamental to their social confidence and reduced self‐stigma. Bodily integrity was the embodiment of a moral integrity, and for men it was also an important signifier of a rejuvenated hegemonic masculinity, an embodiment of their ability to work and provide for their family again after sickness and dependence on others.

One might anticipate widening patterns of disclosure because of ART, as PLWH look healthy and self‐stigma declines (Bond 2009). However, anticipated stigma was a prominent theme, revealed by narratives about concealment and the risks and fears of telling others. This reflected both continuing levels of self‐stigmatisation (fear of gossip and visible side‐effects), as well as fear of enacted stigma (rejection and ‘HIV being used against you’). Most participants carefully managed their disclosure, and one subgroup, mainly men, adopted a strategy of almost complete secrecy. A deep distrust remained about how people would think and react if told. In a few words disclosure can change and fix your new and spoiled identity permanently (Bond 2009). Despite some degree of normalisation of HIV in the era of ART, HIV was therefore not quite ‘just like any other disease’ when it came to telling people (McGrath *et al*. 2014, Roura *et al*. 2009a).

Anticipated stigma has been found in other studies from the region (Mbonye *et al*. 2013, Roura *et al*. 2009a, 2009b). We interpret anticipated stigma as a strong indication that structural or institutional processes of stigmatisation are still operating in this setting, and structures of gender were influencing patterns of anticipated stigma. Women in general experienced all the forms of continuing and changing stigma documented in this article: a degree of continuing self‐stigma, anticipated stigma in terms of fear of gossip and visible side‐effects, and a fear of enacted stigma if people were to discover their HIV status. In contrast, men in general focused their fears on heightened self‐stigma, related to gossip and their masculine identity and dignity in the community being undermined.

Women's greater anticipation of enacted forms of stigma reflected a continuing and powerful discourse that morally judges, blames and punishes women more harshly than men for their HIV status, particularly more marginalised, poor women who face many forms of stigmatisation (Castro and Farmer 2005). Evidence from other studies in Africa shows that women are more likely to experience or fear enacted stigma, whereas men talk more about self‐stigmatisation and their fears about how the community might perceive them or gossip about them (LeClerc‐Madlala 2001, Shamos *et al*. 2009, Simbayi *et al*. 2007, Wyrod 2011).

Fear of rejection among the women reflected their fear about their partner's judgement of their moral integrity and being labelled as promiscuous or a prostitute. Harsher stigmatisation of women who are labelled as bad women or prostitutes because of their HIV status are the products of patriarchy and specific constructions of moral and immoral femininity in this setting (Davis 2000, Ogden 1996).

In contrast, men feared gossip more than they feared rejection. One of their priorities was to be seen by their neighbours and family as a proper man who was strong, able to work and provide for his family. HIV stigma was therefore reinforcing existing notions of hegemonic masculinity (Wyrod 2011). Men felt that their identity as a proper man, and the reduced self‐stigma they felt after recovery, was fragile and highly dependent on the community or their work peers not knowing they were HIV positive. Other research in the region also reports the significance of internalised stigma for men (Lynch *et al*. 2010, Poku *et al*. 2005, Simbayi *et al*. 2007, Shamos *et al*. 2009).

Applying Phelan *et al*.'s (2008) functions of stigma, our findings show that gossip was described as a form of surveillance to identify and label others, both for norm enforcement and disease avoidance functions. Sociological analysis argues that gossip allows groups to reinforce their own sense of moral normality and righteousness compared to an immoral community (Ogden and Nyblade 2005, Phelan *et al*. 2008), that gossip is ‘one of the chief weapons which those who consider themselves higher in status use to put those whom they consider lower in their proper place’ (Gluckman 1963: 309).

Women's fear of their HIV status being used against them also indicated the power and domination function of stigma at the micro‐level (Phelan *et al*. 2008). Through norm enforcement and domination, stigma could be seen to be reproducing gender hierarchies by condemning women and men who stray from dominant notions of what constitutes a proper woman or man.

By making people look better, participants argued that ART had also generated a new motive for surveillance by the community, because PLWH could no longer be physically identified, but still remained a ‘threat.’ There was a need to look for the signs more carefully through observation, hearsay and gossip, a new aspect of stigma also identified by Roura *et al*. (2009b).

Our findings show that ART is not eliminating the underlying structural causes of stigma and certain manifestations of stigma. Continuing stigma sustains barriers to HIV testing (Roura *et al*. 2009b) and has implications for the long‐term self‐management of HIV, especially in inhibiting disclosure in new intimate relationships (Mbonye *et al*. 2013), which then exposes people to adherence difficulties and increases the risks of new infections.

The findings show the vital role of health workers in reducing stigma, and that ART programmes need to sustain counselling and support to people about dealing with stigma and disclosure. They also revealed that men and women are using their agency to challenge stigma, at interpersonal and intra‐personal levels, through resistance thinking and everyday acts of support to others with HIV, processes strengthened by a sense of collective identity and solidarity with their peers. The findings also reiterated the importance of peer‐led support that can help men and women talk though HIV‐related problems affecting their self‐esteem, to tackle self‐stigma, reconstruct alternative, positive identities and enhance wellbeing (Igonya and Moyer 2013, Wyrod 2011).
